# System transferability of Raman-based oesophageal tissue classification using modern machine learning to support multi-centre clinical diagnostics

**DOI:** 10.1038/s44276-024-00080-8

**Published:** 2024-07-23

**Authors:** Nathan Blake, Riana Gaifulina, Martin Isabelle, Jennifer Dorney, Manuel Rodriguez-Justo, Katherine Lau, Stéphanie Ohrel, Gavin Lloyd, Neil Shepherd, Aaran Lewis, Catherine A. Kendall, Nick Stone, Ian Bell, Geraint Thomas

**Affiliations:** 1 Department of Cell and Developmental Biology, University College London, Gower Street, London, WC1E 6BT UK; 2 Translational Sciences, Adaptimmune Therapeutics Plc, Jubilee Avenue, Abingdon, OX14 4RX UK; 3 Biomedical Physics, Department of Physics and Astronomy, University of Exeter, Stocker Road, Exeter, EX4 4QL UK; 4 Department of Research Pathology, Cancer Institute, University College London, Gower Street, London, WC1E 6BT UK; 5 Spectroscopy Products Division, Renishaw PLC, New Mills, Wotton-under-Edge, GL12 8JR UK; 6 Biophotonics Research Unit and Pathology Department, Gloucestershire Hospitals NHS Foundation Trust, Great Western Rd, Gloucester, GL12 8JR UK; 7 Gloucestershire Cellular Pathology Laboratory, Cheltenham General Hospital, Sandford Road, Cheltenham, GL53 7AN UK

## Abstract

**Background:**

The clinical potential of Raman spectroscopy is well established but has yet to become established in routine oncology workflows. One barrier slowing clinical adoption is a lack of evidence demonstrating that data taken on one spectrometer transfers across to data taken on another spectrometer to provide consistent diagnoses.

**Methods:**

We investigated multi-centre transferability using human oesophageal tissue. Raman spectra were taken across three different centres with different spectrometers of the same make and model. By using a common protocol, we aimed to minimise the difference in machine learning performance between centres.

**Results:**

61 oesophageal samples from 51 patients were interrogated by Raman spectroscopy at each centre and classified into one of five pathologies. The overall accuracy and log-loss did not significantly vary when a model trained upon data from any one centre was applied to data taken at the other centres. Computational methods to correct for the data during pre-processing were not needed.

**Conclusion:**

We have found that when using the same make and model of spectrometer, together with a common protocol, across different centres it is possible to achieve system transferability without the need for additional computational instrument correction.

## Background

Raman spectroscopy (RS) is a technique that is able to detect the vibrational modes of a molecule. A Raman spectrum is unique to the inelastically scattered photons from a given molecule and can be used to identify the biochemical constituents of a biomedical sample. Unfortunately, biomedical samples are highly complex, resulting in many overlapping Raman signals, requiring computational methods to interpret the underlying biochemical features. This must be done in the presence of various sources of non-Raman signals emanating from the laser-sample interaction, from cosmic rays and components of the instrument itself. These can overwhelm the relatively weak Raman effect. Improvements to Raman spectrometers and computational methods have made this task tractable, and many proof-of-concept studies demonstrate that RS can be applied to cancer samples in order to; classify cancers from tissue or other biological samples (e.g. serum) [1–3], to detect pre-cancerous changes [4, 5] and to detect tumour edges during surgery [6, 7]. Oesophageal cancer diagnostics is a setting that could gain much from the clinical translation of RS. Oesophageal cancers are often diagnosed late, resulting in high mortality rates. Pre-cancerous lesions are difficult to identify during endoscopy and the histopathological reliability of excised tissue is known to be problematic [8, 9]. In particular, diagnoses based on morphological information are known to be limited, with kappa statistics of agreement amongst pathologists as low as 0.28 [10]. There are a number of complementary techniques, such as immunohistochemistry (IHC), which can increase this agreement. However, this technique is not without its drawbacks. For instance, p53 immunostaining, a strong prognostic marker in oesophageal cancers, is only interpretable in approximately 80% of cases [10]. There is evidence that RS may be able to further augment such information. A recent meta-analysis found that ex vivo applications of RS to diagnose oesophageal cancers had a pooled Area Under the Receiver Operating Characteristic (AUROC) curve of 0.99 [11]. Such results establish the potential of RS to discriminate between healthy and cancerous samples. However, there are a number of issues hindering its ability to generalise to the clinical setting, including small sample sizes, biased validation strategies, simpler binary datasets, and sub-optimal pre-processing [3]. In particular, these studies were performed at single centres and the transferability of a model developed at one centre to unseen clinical settings has yet to be established.

This issue occurs because Raman spectrometers are high-precision instruments, and slight variations in any of their components can result in deviations in Raman wavenumber and intensity values [12, 13]. There are a number of components that contribute to these deviations, including the excitation source, wavelength selector, detector, and optics [14]. In addition, datasets may be obtained under different conditions, such as differing acquisition times or laser powers, resulting in heterogeneous datasets. These variations are especially problematic in biomedical samples which often only manifest marginal differences between disease classes. Additionally, machine learning (ML) approaches which utilise wavenumber selection are particularly prone to poor generalisation due to their sensitivity to shifts in the wavenumber axis [15].

The attempt to make a model trained on one spectrometer applicable to other instruments has been called model transfer. To this end, two main subdivisions have been described in the literature: spectral standardisation and model updating [15]. The former adjusts datasets to make them more similar by, for instance, calibrating their wavenumber axes to align with prominent Raman peaks. It can also include instrument response correction to remove the effect of detection efficiency varying with wavenumber. Such perturbations are known to deteriorate ML performance [16]. Spectral standardisation methods have been shown to ameliorate between-system deviations [17, 18]. Model updating seeks to make the ML models themselves robust to variations between datasets. This can be achieved by training models with data from multiple instruments, thus forcing a model to be robust against dataset variations. It could also be achieved by selecting shared and invariant features between datasets. Model updating by training traditional ML models, such as Partial Least Squares, on data from multiple instruments has been found to be ineffective [15]. However, the recent utilisation of deep learning (DL) in RS applied to oncology samples may provide a solution [3, 19, 20]. Convolutional neural networks (CNN), first developed in the context of image recognition, are known to be able to learn invariance to a number of input perturbations: regardless of the position of an object in an image, even if rotated, the model can still accurately recognise the object. In the context of biomedical RS, invariance to translation, stretching, and intensity may make a model robust to model transfer.

In this study, we expand upon the work of Isabelle et al., who investigated the system transferability of an oesophageal dataset taken across three centres [21]. They found that although instrument calibration methods resulted in a demonstrable concordance of data across three centres, this did not translate to an improvement in binary classification performance when transferring models between systems. Here we expand the sample size and the number of classes while also exploring a greater range of modelling techniques, namely Principal Component Analysis - Linear Discriminant Analysis (PCA-LDA), Support Vector Machine (SVM), and CNN.

## Methods

### Sample collection and preparation

Samples were obtained from patients with a scheduled endoscopy for Barrett’s surveillance or from patients who had surgery for oesophageal cancer (Fig. SI1). Barrett’s oesophagus involves metaplastic changes to the columnar mucosa and can predispose individuals to oesophageal cancer [22]. 66 FFPE samples from 51 patients were taken from the histopathology archive at Gloucestershire Hospitals NHS Foundation Trust, from which patients had given informed consent for their tissues being used for future research. These procedures were performed under local (endoscopic resection) or general (oesophageal resection) anaesthetic in accordance with an approved ethical proposal [Gloucestershire Local Research Ethics Committee]. Routine histopathology reports were used to assist with sample selection, while clinical data managers helped identify representative regions with one distinct pathology, identifying one of five clear histologies: normal squamous (NSQ), intestinal metaplasia (IM), low and high-grade dysplasia (LGD and HGD) and adenocarcinoma (AC). Being fully accredited by UKAS, all samples adhered to ISO15189 standards. 8 μm tissue sections were cut and mounted onto stainless steel slides as described in detail in SI2. Three contiguous sections were collected from each sample and one was sent to each of the three participating centres (Biophotonics Research Unit, Gloucestershire Hospitals NHS Foundation Trust, Gloucester, UK; University of Exeter, Exeter, UK; and University College London, London, UK). Standard H&E slides of adjacent tissues were also taken to identify regions of interest and to confirm pathologies. One consultant and one registrar histopathologist outlined regions of interest and confirmed diagnosis.

Each centre used the same make of benchtop spectrometer; prototype Renishaw RA816 Biological Analyser (Renishaw plc, Wotton-under-edge, UK). These are configured for pathology use with a 785 nm laser excitation, a 50x NA 0.8 objective, a 1500 mm^−1^ grating, and a motorised XYZ stage. Data was collected in StreamLine^TM^ mode over the fingerprint region 400−2200 cm^−1^, using a 10 μm grid and an integrated exposure time of 6 seconds per point. Data was collected over spatial regions exhibiting a homogenous pathology, with map sizes varying from 11 × 18 to 75 × 93 spectra. All three centres followed the same protocol for taking the Raman maps. Regions of interest were matched between all centres to that identified by the histopathologists so that all centres mapped approximately the same regions: this was not exact as the slides were taken from adjacent sections of the same tissue. Samples that breached the protocol at any centre were removed from the downstream analysis, leaving 61 samples from 51 patients.

### Preprocessing

Cosmic rays were removed using 3 × 3 spatial median filtering across adjacent spectra. Saturated spectra were automatically flagged by the manufacturer’s software and removed. This resulted in 560819 spectra, unevenly distributed over the five classes (Fig. SI3). Each spectrum was standard normal variate normalised.

### Machine learning and cross-validation strategy

#### Model development

PCA-LDA is a common modelling technique in biomedical applications of RS for cancer diagnostics [3]. In particular, it has been applied for the detection of oesophageal cancer by several groups [13] and therefore provides a useful measure of baseline performance for this study. PCA is used to reduce the dimensionality of a dataset and LDA then constructs a decision boundary in PC space to classify spectra as belonging to one of the five classes. SVMs also construct a decision boundary, but are able to do so in a non-linear fashion by projecting the data into a higher dimensional space. This is achieved using a kernel function: we use the radial-basis function (RBF). These represent ’traditional’ ML models in contrast to ’deep’ learning models.

One type of DL architecture, the CNN, has seen great success in several domains of medical imaging [23], and has become popular in oncological applications of RS [3]. They are able to construct non-linear decision boundaries, allowing them to capture pertinent features from data. It has even been suggested that they negate the need for several pre-processing steps in RS [24]. However, their suitability to medical applications of RS is debated, as the technique typically requires large amounts of data which is often prohibitively expensive to collect in the medical domain and can consume extensive computational resources for training. In the context of DL, this study has very few samples - although there are a copious number of spectra, they are derived from relatively few independent samples. With such a large parameter space that characterises such models and a small sample size, over-fitting becomes a significant risk. It is unclear in this situation whether DL would provide any benefit compared to the traditional ML models.

To assess this, we developed a custom CNN. Although more performant CNNs exist, we constructed a relatively simple CNN to reduce the risk of overfitting, using Python version 3.10 and the Pytorch framework [25]. Model details in SI4. The Scikit-learn library was used for the PCA-LDA and SVM models [26].

#### Instrument calibration and corrections

The reproducibility of wavenumber calibration between the three instruments was estimated to be within 0.5 cm^−1^ across the analysis region, while the reproducibility of system response was estimated to be within 14% before calibration and 5% after (SI5.1). Response calibration data was collected every six months through the manufacturer’s software by employing a NIST SRM 2242 response calibration standard. Data was not response calibrated at source, but instead was optionally applied post-collection so that its effect on model performance could be investigated. All instruments had nominally identical Nikon TU Plan Fluor 50X objectives. Though these were selected to minimise objective contribution to the Raman signal, it remains a significant signal component when collecting in a low signal regime such as tissue sections. Objective reference spectra were collected for all systems while focused on a stainless-steel slide and showed up to 30% difference in shape across the analysis region (SI5.2). The observed maximum objective contribution over the wavenumber region was at 1350 cm^−1^. The median objective signal was estimated to contribute 12% to the signal in this region. Correction for the objective function was performed by including objective spectra for the system in question as a reference component in the extended multiplicative signal correction (EMSC) processing of the data. These are standard instrument correction techniques [27]. To assess the effect of instrument variation on model performance, cross-validation (CV) was performed for all three ML models, with and without response calibration plus objective correction (henceforth referred to as instrument correction).

#### Cross-validation strategy

Overfitting is a well-known problem in ML, in which a model fits so well to the training set that it is modelling random perturbations as opposed to learning real features [3]. This is increasingly becoming recognised in the medical ML literature as a key reason for the ‘performance gap’, whereby excellent research results fail to generalise to clinical settings [28]. A less well-known form of overfitting can occur at the second level of inference: over-optimising model hyperparameters [29, 30]. Hyperparameters are choices that researchers make regarding certain characteristics of a model; for instance, the number of PCs to retain in PCA-LDA, the degree of decision boundary curvature in SVM-RBF, or the learning rate for a CNN. A search for the best-performing hyperparameters risks finding those values which happen to give good performance on the test data, but fail to generalise [31]. This is exacerbated by low sample sizes, as methods to ameliorate this effect, such as nested cross-validation, are only effective when the sample is of sufficient size to allow the validation set to remain representative of the target population. We circumvent this by selecting model hyperparameters based on findings from previous work [32]. As the hyperparameter space was not systematically searched we are gaining generalisability as there can be no overfitting at the second level of inference [31] at the cost of perhaps not finding the optimal model (underfitting). The purpose of this study is to assess how well data were taken at one centre transfers across to other centres. This addresses the problem of how data taken at a single clinical site might be used to construct models that are clinically relevant to other sites. Therefore, we trained our models iteratively one centre at a time, leaving out two centres for testing. For each training centre we performed 3-fold CV. This provides three estimates of performance, sequentially using two-thirds of the data for training and one-third for testing. This was repeated five times, each time with a different random seed so that the training/test splits were different. This process resulted in a total of 15 estimates of model performance. We report the mean. Unfortunately, it is difficult to construct confidence intervals as the data between folds is not independent. We therefore only report the standard deviation. This simulates the process of internal validation, in which performance metrics are obtained by using portions of the existing dataset in lieu of independent (external) datasets. This provides an estimate for how the model will generalise across to the data taken at the other centres. Once this estimate is obtained we then train the entire centre-level dataset and test against the two left-out centres. In this way, we simulate the acquisition of external datasets, albeit from ostensibly identical samples. Additionally, we trained each model on the all-centre dataset, ignoring the centre-level structure, using the same 5 × 3 CV strategy used for the single centres (Fig. SI6). We stratified all CV splits such that class proportions were approximately preserved. It has been shown that when splitting Raman data with a hierarchical structure the split should be performed at the highest level, else spectra from the same sample will be present in both the training and test sets, which artificially inflates performance estimates [3, 33]. Hence, the data was split at the patient level. Additionally, any pre-processing performed using global statistics was done after the data was split to avoid data leakage. The average number of spectra per sample was 3065. In this application, we are interested in the overall sample label, as opposed to the label of individual spectra (unlike margin detection which would require demarcation of the individual spectra within each Raman map). Thus we classified a sample based on a simple consensus amongst all spectra from a sample. In this manner, all spectra are used for model construction, and a single classification per sample is obtained.

Results are reported as accuracy and AUROC. Accuracy is a simple and intuitive metric, but lacks nuance. The AUROC is commonly used in medical diagnostics studies, showing the trade-off between sensitivity and specificity, although it is only defined in binary terms, necessitating a one vs all other classes approach to multiclass classification. Both of these metrics require choosing a threshold above which a sample belongs to one class and below to another class. Such classification metrics are described in the statistical literature as ’improper’ in the sense that they do not capture the predictive ’confidence’ of a model - a value close to the threshold and a value far from it gives the same prediction [34]. The log loss is a ’proper’ scoring metric that is able to capture these subtleties, resulting in a more subtle, though less intuitive, performance metric [35].

### Model sensitivity to instrument perturbations

Deliberately perturbing ML inputs during testing is becoming an increasingly recognised method to assess modes of failure, which helps assess the suitability of a model to the clinical setting [28]. Wavenumber perturbation (peak shifting) and instrument differences induced by the objective lenses and instrument response are some characteristics deemed important during model transfer between Raman instruments [17]. These have been explored in the context of traditional ML techniques [16]. To assess the extent to which perturbations to these components contribute to degrading model performance we performed three in silico experiments.

Three potential sources of between instrument perturbations were simulated, each simulated perturbation being systematically increased. It is expected that at increasing levels of perturbations, model performance will worsen. This can help assess whether any particular model is more robust to such perturbations and gives an estimation of the degree of perturbation that can be tolerated for inter-instrument model transfer.

For all in silico experiments the data from Centre 1 was used, subject to the above-described 5 × 3 CV strategy. Additionally, for every fold, the test data was perturbed in one of three separate ways (detailed below). Thus the training data was a subset of that empirically derived from Centre 1 without any instrument correction. The test data, however, had varying degrees of perturbations induced to simulate the effect of being taken from a different instrument. Note that the purpose of these in silico experiments was not to accurately model instrumental effects, but rather to estimate the extent of different instrument perturbations the ML models may be able to tolerate.

#### Simulating wavenumber perturbation

Wavenumber shifting was induced in the test set by shifting each spectrum by a fixed number of data points. Simulations were performed for shifts of up to 5 data points in either direction. Each data point shift corresponds to approximately 2 cm^−1^, hence the equivalent wavenumber shift range was approximately −10 to 10 cm^−1^.

#### Simulating objective lens perturbation

The objective lens difference spectra between instruments were observed to be complex functions of several broad peaks across the analysis region. To simulate the variation of objective contribution between instruments, a decaying sinusoid was added to the test set (Fig. SI5.3). The amplitude of the sinusoid at its greatest extent was obtained from the observed objective lens contribution of centre 1. This was then scaled between −100% and 100% in increments of 20%. The maximum observed difference in objective contribution was between centres 2 and 3 at 30%.

#### Simulating response perturbation

The response factor is a multiplicative factor applied to the data during response calibration. Instrument response was observed to be approximately linear over the range 400–1800 cm^−1^. The response perturbation was simulated to be a line passing through 1 at the centre of the wavenumber region and varying by between 1-p/200 at one end of the region and 1+p/200 at the other, where p is the percentage variation across the data, which varied in the range −100% to 100%. Thus, a series of lines were created to emulate this process with varying degrees of slope in order to simulate the effect of greater and lesser response contributions. The maximum observed difference in response was between centres 1 and 3 with a slope factor of approximately 15%.

## Results

### Data description

**Fig. 1:**
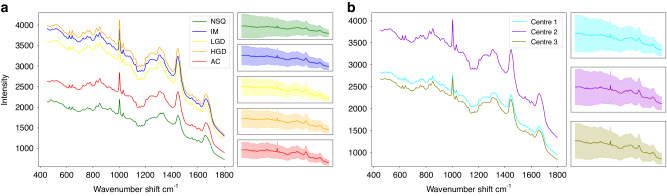
Average spectra for the unnormalised and uncorrected data. **a** Average spectrum by class. **b** Average spectrum by centre. Right hand panels show 1 SD.

Fig. 1a shows the average spectrum per class across all centres. Fig. 1b shows the average spectrum per centre. It is difficult to draw conclusions from these graphs alone, but it is apparent that the three intermediary pathologies of IM, LGD, and HGD have, on average, a greater intensity than the more distinct groups of NSQ and AC. Similarly, spectra collected from centre 3 has a generally higher intensity. This effect disappears during normalisation.

### Wavenumber perturbation results

**Fig. 2:**
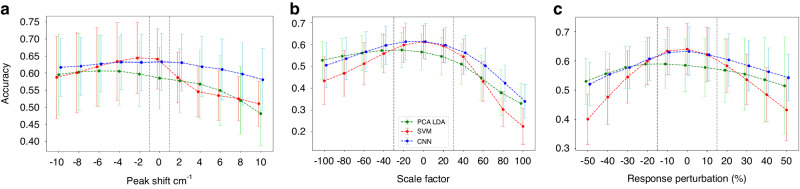
Perturbation accuracy of the test sets using PCA-LDA (green), SVM (red), and CNN (blue) via three separate perturbation methods. **a** Effect of applying wavenumber shift perturbation (cm^−1^). **b** Effect of applying the objective perturbation, given as a percentage of the total estimated objective contribution that was added. **c** Effect of applying the response perturbation (%), given as the percentage change induced across the data. Mean accuracies are reported +/− 1 SD. Black dotted lines correspond to the maximum response perturbation observed between the three instruments (detailed in SI5).

The maximum wavenumber shift measured between the three study instruments was 0.5 cm^−1^. Fig. 2a shows that the CNN and PCA-LDA models are robust to wavenumber perturbation within this observed range. The CNN performance remains robust beyond these limits. The SVM performance is particularly sensitive to this source of perturbation.

### Objective lens perturbation results

Varying the additive contribution of a simulated objective lens largely results in a gradual and smooth deterioration of performance over the simulated range. The maximum observed difference in objective signal contribution between the three centres in this study was approximately 30%. This is indicated in Fig. 2b as black dotted lines. Beyond this range, the performance of all the models deteriorates, but again the SVM performance is particularly precipitous.

### Instrument response perturbation results

Varying the multiplicative contribution of a simulated instrument response also gives a gradual and smooth deterioration of performance over the simulated range (Fig. 2c). The black dotted lines show the range of estimated perturbations observed between the three study instruments. Within that range, all models are reasonably robust.

### Simulated perturbations

Overall, the simulated perturbation results suggest that these models are robust over some range of contributions from these three sources of instrumental differences. For all simulations, this range is greater than the measured instrument-to-instrument differences, and therefore no significant impact on model performance is expected when transferring between instruments. The extent to which these perturbations can be tolerated is model-dependent. The trends from the results suggest the SVMs are more sensitive, especially with larger perturbations, consistent with the findings of Sattlecker et al. [16]. The CNN is more robust across a larger range of perturbations for all perturbation types. This is consistent with the invariance property of CNNs. However, the overlap in performance between CV folds, evident in the large standard deviations, necessitates caution in this interpretation. It may be possible to further exploit this property by inducing these perturbations in the training set in a process known as data augmentation, which was not explored here.

### System transferability

**Fig. 3:**
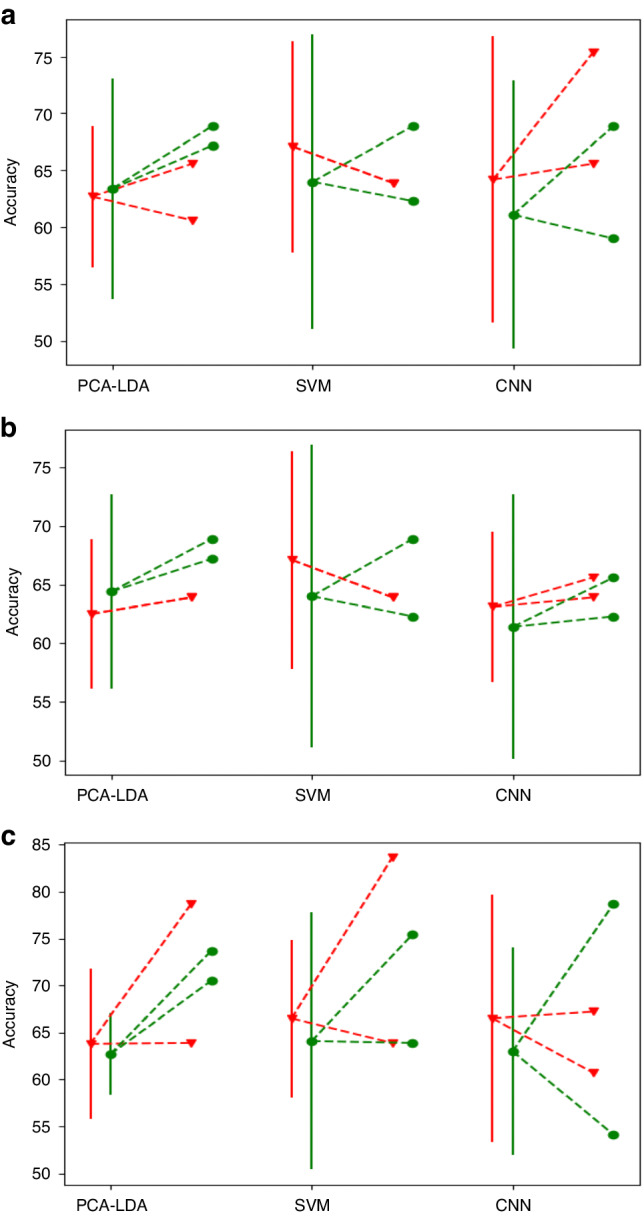
Uncorrected vs corrected accuracy comparison. Dots with solid lines indicate single centre 5 × 3 CV mean accuracy +/− 1 SD with dashed lines to dots indicating test centre accuracies. Red triangular dots indicate uncorrected data, and green circular dots indicate corrected data. **a** Training on Centre 1. **b** Training on Centre 2. **c** Training on Centre 3.

Tables 1 and 2 compare the uncorrected and instrument-corrected dataset performances. In order to more easily visualize how these compare, the change in performance from the 5 × 3 CV score per centre to the two held-out test centres is shown in Fig. 3. These show there is no appreciable drop in the training accuracy to that of the test centres as would be expected if transferability was problematic. This is consistent with the simulation results, in which the range of perturbation which adversely affects performance was greater than that observed between the instruments. Additionally, according to the accuracy, there is little to distinguish the performance of the three models on either dataset. The log loss, however, suggests possible superiority of the CNN, giving marginally lower scores, and with much narrower variability, indicating the model gives a more robust estimate of performance (Table SI7.1).

**Table 1:** Uncorrected data results per model.

		5 × 3 CV one centre	Centre 1	Centre 2	Centre 3	5 × 3 CV All centres
**PCA-LDA**	Centre 1	63.8% +/− 8.0	*72.1%*	63.9%	78.7%	64.2% +/− 6.9
	Centre 2	62.5% +/− 6.4	63.9%	*70.5%*	63.9%	
	Centre 3	62.7% +/− 6.2	65.6%	60.6%	*68.9%*	
**SVM**	Centre 1	66.5% +/− 8.4	*75.4%*	63.9%	83.6%	63.9% +/− 11.2
	Centre 2	67.1% +/− 9.3	63.9%	*77.0%*	63.9%	
	Centre 3	66.5% +/− 10.7	67.2%	63.9%	*75.4%*	
**CNN**	Centre 1	66.5% +/− 13.2	*100%*	67.2%	60.7%	68.4% +/− 5.6
	Centre 2	63.1% +/− 6.4	65.6%	*90.2%*	63.9%	
	Centre 3	64.2% +/− 12.6	75.4%	65.6%	*91.8%*	

Top line per centre represents accuracy (%) +/− 1 SD, bottom line represents log loss +/− 1 SD. *Italicised* entries indicate the training results and so are not indicative of performance.

**Table 2:** Corrected data results per model.

		5 × 3 CV one centre	Centre 1	Centre 2	Centre 3	5 × 3 CV All centres
**PCA-LDA**	Centre 1	62.7% +/− 4.3	*70.4%*	70.5%	73.7%	62.2% +/− 11.2
	Centre 2	64.4% +/− 8.3	67.2%	*75.4%*	68.9%	
	Centre 3	63.4% +/− 9.7	68.9%	67.2%	*73.8%*	
**SVM**	Centre 1	64.1% +/− 13.7	*80.3%*	63.9%	75.4%	66.6% +/− 8.8
	Centre 2	64.0% +/− 12.9	62.3%	*80.3%*	68.9%	
	Centre 3	65.1% +/− 12.1	62.2%	65.6%	*78.7%*	
**CNN**	Centre 1	63.0% +/− 11.1	*100%*	54.1%	78.7%	68.2% +/− 9.6
	Centre 2	61.4% +/− 11.3	62.3%	*100%*	65.6%	
	Centre 3	61.1% +/− 11.8	68.9%	59.0%	*93.4%*	

Top line per centre represents accuracy (%) +/− 1 SD, bottom line represents log loss +/− 1 SD. Italicised entries indicate the training results and so are not indicative of performance.

### Model performance

As there is no discernible difference in performance between the uncorrected and instrument-corrected data we proceed in the most parsimonious manner; with the uncorrected data. Due to the unintuitive interpretation and relative lack of use of the log loss in biomedical studies, we focus on AUROC in the proceeding discussion. A meta-analysis of RS applied to ex vivo oesophageal cancers found a pooled AUROC of 0.99 when distinguishing between malignant and benign tissues, indicating that RS has potential for use in distinguishing malignant oesophageal pathologies [11]. Our results accord with this (Table 3). SVM and CNN clearly have superior performance. SVM is better at discriminating AC samples (AUROC of 0.97 vs 0.91), but this comes with a trend of less discriminatory power over the intermediate classes of IM, LGD, and HGD. Which model is preferable depends upon the clinical consequences of such misclassifications. Unfortunately, histopathological definitions vary worldwide, as do clinical pathways [36]. In LGD, molecular architecture is largely preserved with only subtle morphological changes, making it difficult to distinguish from non-dysplastic tissue [37]. HGD displays more marked atypical features and hence different morphological features. However, there is no clear demarcation between the two, leading to high inter and intra-rater variability [8, 38]. The distinction between grades is clinically important as their management options vary. LGD is managed via endoscopic surveillance with endoscopic resection as needed, or potentially with radiofrequency (RFA) ablation. Patients with HGD undergo more intensive surveillance with options including RFA +/− endoscopic mucosal resection and surgical resection. The clinical relevance of IM is less clear. IM is characterized by the presence of goblet cells, normally present in the intestine. It is associated with a risk of progressing to LGD [37]. However, its clinical relevance is dependent upon its location in the oesophagus [37]. This is further complicated by the fact that IM can be further subdivided into three types, one of which is not deemed a risk factor for gastric cancer, and the remaining two having an association with developing cancer, but with an unclear causal pathway [39]. There is debate in the community regarding the necessity of the presence of IM for the diagnosis of Barrett’s oesophagus [40].

**Table 3:** Uncorrected data: Class specific AUROC + /− 1 SD by model.

	NSQ	IM	LGD	HGD	AC
PCA-LDA	0.99 + /− 0.01	0.78 + /− 0.08	0.69 + /− 0.11	0.51 + /− 0.10	0.74 + /− 0.08
SVM	0.99 + /− 0.01	0.87 + /− 0.09	0.89 + /− 0.08	0.89 + /− 0.08	0.97 + /− 0.02
CNN	0.99 + /− 0.00	0.90 + /− 0.06	0.92 + /− 0.08	0.92 + /− 0.04	0.91 + /− 0.08

Few oesophageal RS studies have attempted to distinguish between pathology sub-types; those that have found performance over the intermediary classes worse than NSQ vs all [41]. As RS moves from proof-of-concept to clinical deployment, more studies will need to focus on increasing the discriminative ability between intermediary classes. DL architectures have elsewhere shown potential to this end [3].

## Discussion

The single centre 5 × 3 CV training process simulates the process of how data would be collected and trained: iterative subsets of the centre data would be used to train a model and the remaining centre data would be used to estimate the ability of the model to generalise to unseen data. As ML is a data-driven approach, it is sensitive to the training data, which manifests in the variation of performances across folds summarised by the standard deviation. When the models are subsequently trained on the full centre data, this simulates the process of model deployment. Finally, by testing against the held-out centres, we simulated the process of testing the model on unseen data. The caveat is that we used near identical samples, thus we can be confident that any differences in performance are due solely to (unwanted) instrument effects, as opposed to being an artefact of the data. Any deviations due to protocol interpretation by the different operators across the centres were sufficiently minimal to allow the transfer. As the centre level test results are within the expected range of performance as estimated by the single centre level 5 × 3 CV, we can conclude that our models trained on a single centre transfer well across to data taken on the same make and model of spectrometer when a common protocol has been followed. Although this is a more limited arrangement than establishing transferability across disparate spectrometers and protocols, it establishes the possibility of developing RS models between centres for targeted clinical applications. This would necessitate the development of protocols suitable for each institution and sufficient training of staff. By applying RS to FFPE tissues, we largely adhered to current pathology pathways, only diverging by taking an additional section for mounting onto stainless-steel slides. Once an additional dewaxing step has been taken, the sections are ready for Raman analysis. However, research workflows would need scaling up to facilitate clinical workflows and high-through-put RS techniques may be necessary to meet clinical demand.

A recent study exploring system transferability across a large selection of spectrometers states that computational methods are urgently needed to render RS models generalisable across settings [16]. Our results suggest that transferability is not a concern between different spectrometers of the same make and model following a common protocol. The in silico simulations suggest that CNNs could provide at least a partial solution, exploiting their input invariance property to provide an alternative means to achieve system transferability. The invariance property could be further leveraged by inducing realistic artefacts during data augmentation. This is a common method in DL which can help models ignore irrelevant features. Although DL comes with its own limitations, we have shown that CNNs are at least competitive with traditional ML models even with relatively small samples sizes.

Although we collected a large number of spectra, these came from relatively few patients which are unlikely to be representative of the entire population of interest. This limits the generalisability of our work and would need to be conducted on a larger cohort to ensure the results extend across the population.

## Conclusions

The results from this study demonstrate that system transferability can be achieved through instrument engineering with clinical protocol adherence alone, bypassing the need for post-data-collection corrections. Additionally, the advantage of CNNs over PCA-LDA and SVMs is suggested and would likely manifest more clearly with larger sample sizes. Although the performance metrics are competitive with existing modalities for oesophageal cancer diagnostics, this would require confirmation with a much larger sample size.

## Supplementary information

Supplementary Information

## Data Availability

Availability of data and materials. The data is available at the Zenodo repository: https://zenodo.org/records/10229090 The protocol is available at the Zenodo repository: https://zenodo.org/records/10405264.

## Code availability

Code is available at GitHub: https://github.com/NBlake/Raman-System-Transferability.
