# Supplementary material for: System transferability of Raman-based oesophageal tissue classification using modern machine learning to support multi-centre clinical diagnostics
Source: BJC Rep. 2024 Jul 23;2:52. doi: 10.1038/s44276-024-00080-8 (PMC11523930; doi:10.1038/s44276-024-00080-8)
Supplement: Supplementary file 1 — Supplementary Information [file 44276_2024_80_MOESM1_ESM.docx]

**Supplementary Information**

This supplementary information file includes figures referenced in the main text and describes details that, while not critical to understanding the overall thrust of the paper, will be beneficial for anyone seeking to replicate the findings.

**Supplementary Information 1**

  
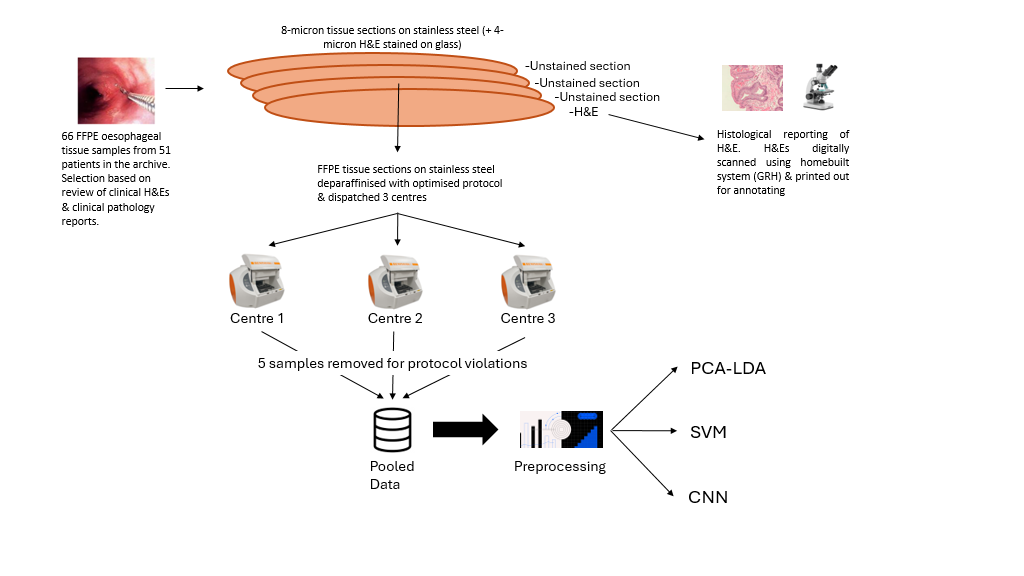


**Supplementary Information 2**


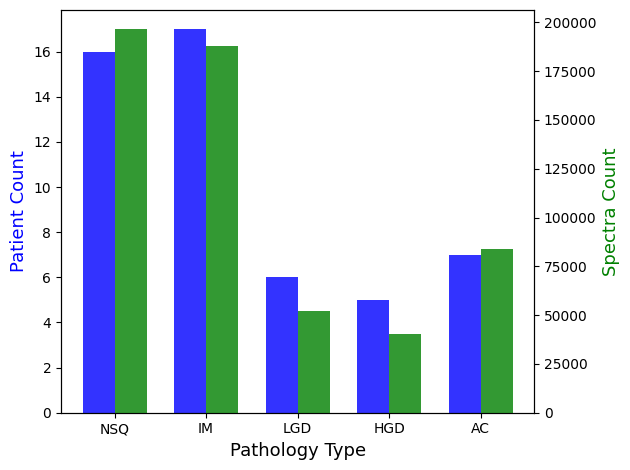


Fig. SI2 Patient and Spectra Count by Pathology Class

**Supplementary Information 3**


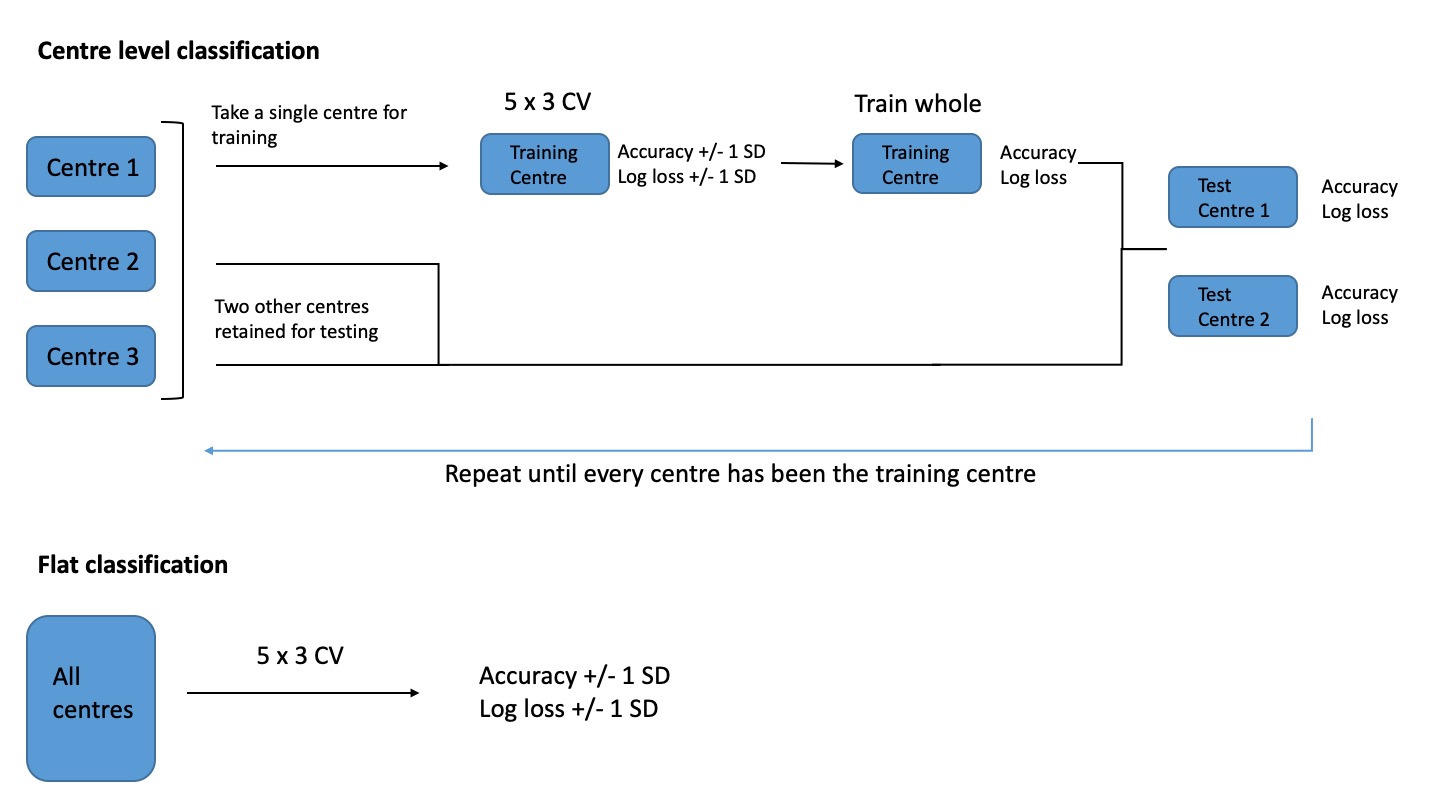


Fig. SI3 CV strategy. Centre level classification first performs 5 x 3 CV to obtain a performance

estimate before training on the whole centre level data, as would be done with a model being deployed in a real setting. This model is then tested against the two held out test centres. Flat classification takes all the data across the centres and performs 5 x 3 CV.

**Supplementary Information 4**


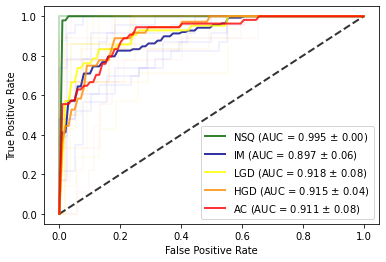


Fig. SI4 CNN ROC curves for each class vs all other classes. Solid lines represent the mean curve per

class, pale lines represent curves from individual folds

**Supplementary Information 5: Slide Substrates**

Tissue undergoing Raman spectroscopy was mounted onto 304L super mirror stainless steel slides. In tissues these have been shown to improve Raman signal acquisition by up to a factor of four and reduce background signal compared to calcium fluoride (CaF2), the standard substrates often used in RS and are far cheaper [42, 43]. Before any tissue was mounted onto the steel slides, they were cleaned by sonication in trichloroethylene for 30 minutes, followed by acetone for 30 minutes then isopropanol for a further 30 min and were then dried under a stream of nitrogen and stored at room temperature.

**Supplementary Information 6: Tissue Processing**

All tissues were received as FFPE blocks. These were manually sectioned using a Leica RM 2235 microtome (Leica Biosystems Ltd., UK) producing adjacent paraffin ribbons of 8m to 3m. These were floated onto a 45◦C water bath and the 8m ribbons mounted onto 304L super mirror stainless steel slides (Renishaw PLC, UK) and the 3m ribbons onto conventional microscopy slides for those samples destined for RS and H&E staining respectively. The mounted steel slides are incubated at 37◦C for 24 hours. The H&E slides were subject to standard automated staining and cover-slipping. Prior to Raman data collection the paraffin, in which the mounted tissues are embedded, needs removing. This was done by immersing the mounted steel slides in four successive ten minute baths in xylene (VWR International Ltd., UK) with gentle agitation. A series of rehydration steps in graded ethanol absolute (VWR International Ltd., UK) took place via two sequential immersions in each of 100%, 90%, 70% and 50% ethanol baths for five minutes each, followed by a final immersion in distilled water for ten minutes.

**Supplementary Information 7: Calibration and Reproducibility**

In the manufacturer’s performance qualification routine, instrument performance optimisation and wavenumber calibration are performed automatically prior to sample data collection, using built-in rare-gas and Silicon standards. Validation spectra are recorded from an internal polystyrene standard as an integral part of this routine. These polystyrene validation spectra were analysed to assess the instrument-to-instrument reproducibility of wavenumber calibration and instrument response. Validation data spanned the period from system installation up to the end of data collection; 10 – 12 months depending on centre. The sample data reported in this study were collected in approximately the last three months of this period.

7.1 Wavenumber Reproducibility

Band centre positions were determined for the three polystyrene bands at 620.9 +/- 0.69, 1001.4 +/-0.54, 1602.3 +/- 0.731 (errors represent one standard deviation) by curve-fitting the bands with a mixed Lorentzian-Gaussian curve model using the manufacturer’s software. The mean and standard deviation of these band positions over time relative to the central ASTM value [44] is shown in figure SI6.1.


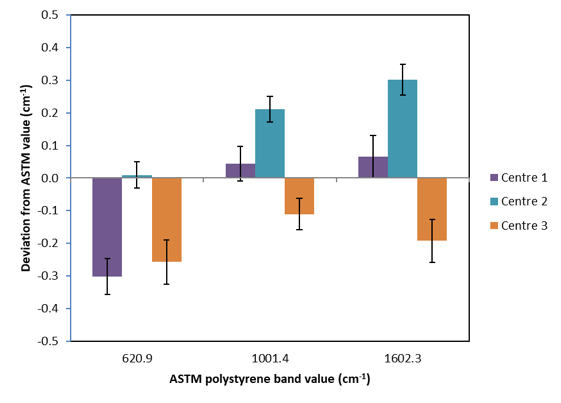


Fig. SI7.1 Polystyrene band positions relative to the ASTM central value shown for all systems. The error bars represent one standard deviation.

7.2 Response Reproducibility

Relative band intensity ratios were obtained for the polystyrene spectra by computing the signal-to-linear-baseline of polystyrene bands integrated over the regions “620” [580, 660], “800” [720, 880], “1030” [930, 1130], “1200” [1130, 1270] and “1600” [1550, 1650] cm-1. The following four ratios were calculated as shape indicators: 620/1600, 800/1600, 1030/1600, 1200/1600. The mean and standard deviation over time of these ratios is shown in figure SI6.2 as percentage deviation from the mean of the ratio across instruments. Ratios are shown before and after response calibration. Response calibration data were collected every six months through the manufacturer’s software by employing a NIST SRM 2242 response calibration standard. For this study the data were not response calibrated at source, but instead the calibration is optionally applied post-collection so that its effect on model performance may be investigated. The reproducibility of system response was estimated to be within 14% before calibration and 5% after.


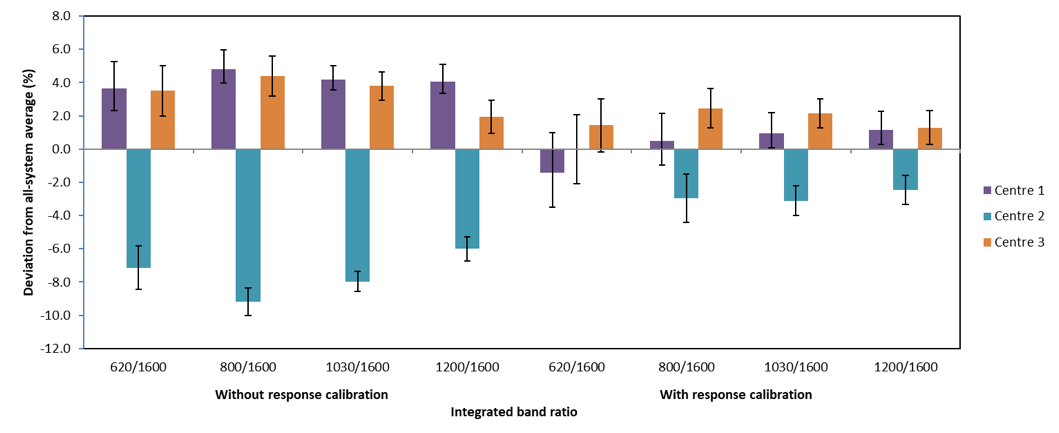


Fig. SI7.2 Percentage deviation of system response from the all-system average measured using polystyrene band ratios. Band ratios are shown for all systems before and after calibration.

7.3 Objective Lens Calibration

All instruments had nominally identical Nikon TU Plan Fluor 50X objectives. Though these were selected to minimise objective contribution to the Raman signal, it remains a significant signal component when collecting in a low signal regime such as from tissue sections. Objective reference spectra were collected for all systems while focused on a clean stainless-steel slide and show up to 30% difference in shape across the analysis region. In section 3.6, correction for the objective function was performed by including these objective spectra for the system in question as a reference component in EMSC-processing of the data. For the objective lens simulation of section 2.4.2, the difference spectrum between objective lens spectra was observed to be approximately sinusoidal, motivating the choice of function to simulate this artefact (fig SI6.3).


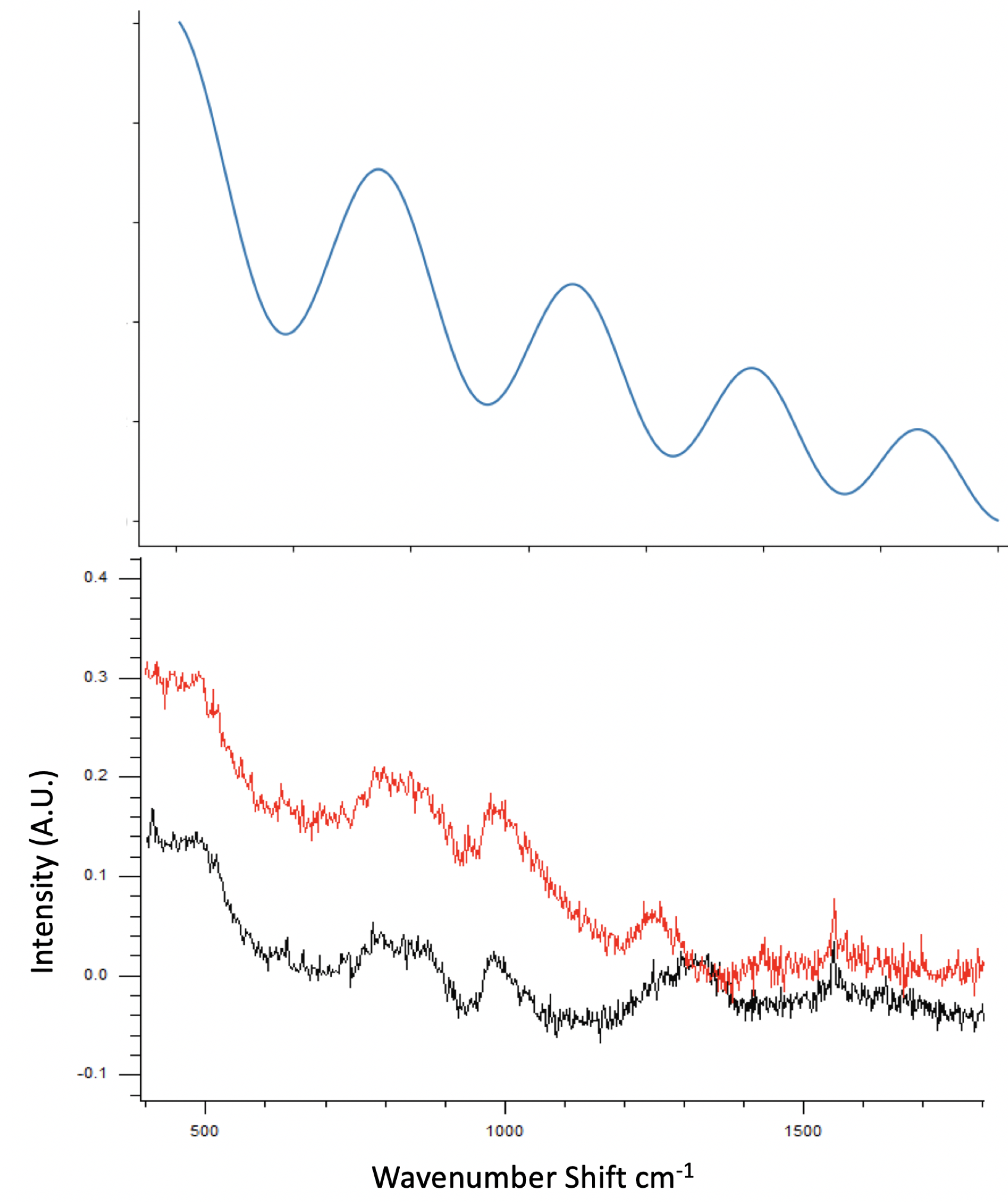


Fig. SI7.3 Top: simulated objective function. Bottom: measured objective difference spectra (normalised)

**Supplementary Information 8: Model descriptions**

A custom CNN was built. Relative to modern CNNs, this network is very small, with approximately 250,000 parameters. ReLU activation functions were used throughout (except the final fully connected layer which used softmax). The Adam optimiser was used for back propagation, using default values. As described in the main text, hyperparameters were selected based on previous research. These were: learning rate = 5x10^−5^, batch size = 184, dropout rate = 0.2, trained for a maximum of 30 epochs with early stopping if there was no improvement in the validation score over 5 epochs. All other hyperparameters, such as kernel sizes and stride can be seen in figure SI7. Hyperparameters for the PCA-LDA were to retain the first 11 principle components for LDA. Hyperparameters for the SVM-RBF were C = 0.9 and γ = 0.1.


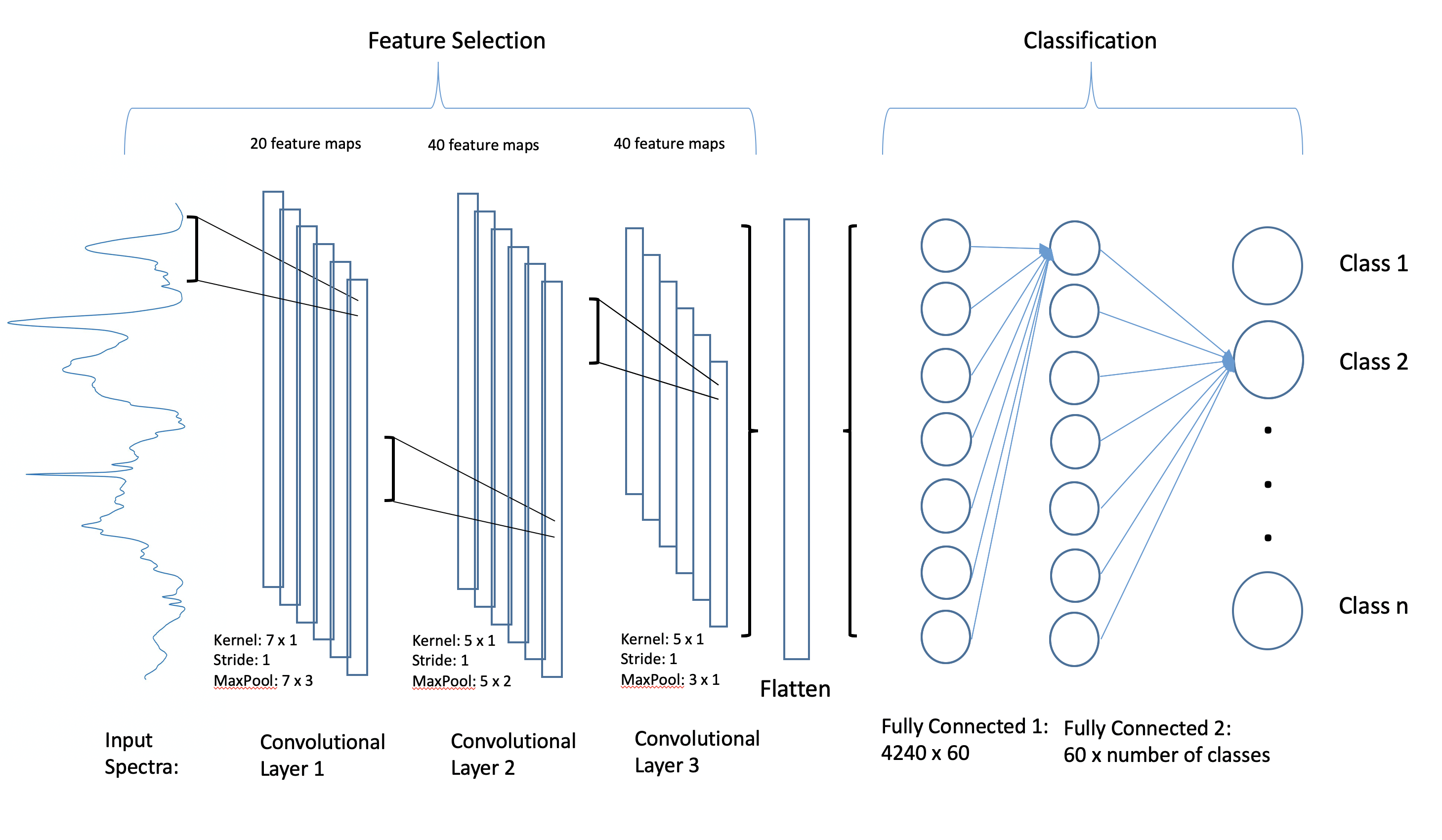


Fig. SI8 CNN architecture

**Supplementary Information 9: Full Results Tables**

These tables are the same as Tables 1 and 2 in the main manuscript but also containing the log loss results

|  | | **5 x 3 CV**  **one centre** | **Centre 1** | **Centre 2** | **Centre 3** | **5 x 3 CV**  **All centres** |
| --- | --- | --- | --- | --- | --- | --- |
| **PCA-LDA** | Centre 1 | 63.8% +/- 8.0  1.76 +/- 0.93 | *72.1%*  *1.03* | 63.9%  2.08 | 78.7%  0.79 | 64.2% +/- 6.9  1.83 +/- 1.01 |
|  | Centre 2 | 62.5% +/- 6.4  1.85 +/- 1.15 | 63.9%  3.56 | *70.5%*  *1.13* | 63.9%  6.12 |  |
|  | Centre 3 | 62.7% +/- 6.2  1.75 +/- 0.85 | 65.6%  1.32 | 60.6%  2.31 | *68.9%*  *1.04* |  |
| **SVM** | Centre 1 | 66.5% +/- 8.4  2.18 +/- 1.99 | *75.4% 0.51* | 63.9%  1.76 | 83.6% 0.64 | 63.9% +/- 11.2  1.63 +/- 1.41 |
|  | Centre 2 | 67.1% +/- 9.3  2.52 +/- 2.21 | 63.9%  3.31 | *77.0%*  *1.22* | 63.9%  4.38 |  |
|  | Centre 3 | 66.5% +/- 10.7 2.19 +/- 1.89 | 67.2% 1.72 | 63.9%  3.55 | *75.4% 0.74* |  |
| **CNN** | Centre 1 | 66.5% +/- 13.2 0.94 +/- 0.26 | *100% 0.29* | 67.2%  0.87 | 60.7% 0.77 | 68.4% +/- 5.6  1.83 +/- 1.01 |
|  | Centre 2 | 63.1% +/- 6.4  1.05 +/- 0.25 | 65.6% 0.70 | *90.2%*  *0.30* | 63.9% 2.37 |  |
|  | Centre 3 | 64.2% +/- 12.6 1.38 +/- 1.08 | 75.4% 0.84 | 65.6%  1.03 | *91.8% 0.30* |  |

Table SI9.1 **Uncorrected** data results per model. Top line per centre represents accuracy (%) +/- 1 SD, bottom line represents log loss +/- 1 SD. *Italicised* entries indicate the training results and so are not indicative of performance.

|  | | **5 x 3 CV**  **one centre** | **Centre 1** | **Centre 2** | **Centre 3** | **5 x 3 CV**  **All centres** |
| --- | --- | --- | --- | --- | --- | --- |
| **PCA-LDA** | Centre 1 | 62.7% +/- 4.3  1.77 +/- 1.06 | *70.4%*  *1.41* | 70.5%  2.73 | 73.7%  1.15 | 62.2% +/- 11.2  1.90 +/- 1.37 |
|  | Centre 2 | 64.4% +/- 8.3  1.78 +/- 0.97 | 67.2% 1.73 | *75.4% 0.61* | 68.9%  1.07 |  |
|  | Centre 3 | 63.4% +/- 9.7  1.85 +/- 1.33 | 68.9%  0.89 | 67.2%  2.44 | *73.8%*  *0.78* |  |
| **SVM** | Centre 1 | 64.1% +/- 13.7  2.04 +/- 1.65 | *80.3%*  *0.39* | 63.9%  1.58 | 75.4%  1.57 | 66.6% +/- 8.8  1.82 +/- 1.31 |
|  | Centre 2 | 64.0% +/- 12.9  1.85 +/- 0.91 | 62.3%  3.41 | *80.3%*  *0.32* | 68.9%  2.81 |  |
|  | Centre 3 | 65.1% +/- 12.1  2.40 +/- 1.93 | 62.2%  3.57 | 65.6%  2.92 | *78.7%*  *0.68* |  |
| **CNN** | Centre 1 | 63.0% +/- 11.1  1.11 +/- 0.52 | *100% 0.19* | 54.1%  1.08 | 78.7%  0.72 | 68.2% +/- 9.6  1.01 +/- 0.23 |
|  | Centre 2 | 61.4% +/- 11.3  1.22 +/- 0.56 | 62.3%  0.93 | *100%*  *0.15* | 65.6%  1.34 |  |
|  | Centre 3 | 61.1% +/- 11.8  1.23 +/- 0.60 | 68.9%  0.87 | 59.0%  1.59 | *93.4%*  *0.19* |  |

Table SI9.2 **Instrument-corrected** data results per model. Top line per centre represents accuracy (%) +/- 1 SD, bottom line represents log loss +/- 1 SD. Italicised entries indicate the training results and so are not indicative of performance.

References.

42. Gaifulina R, Caruana DJ, Oukrif D, Guppy NJ, Culley S, Brown R, et al. Rapid and complete paraffin removal from human tissue sections delivers enhanced Raman spectroscopic and histopathological analysis. Analyst. 2020;145:1499–510.

43. Lewis AT, Gaifulina R, Isabelle M, Dorney J, Woods ML, Lloyd GR, et al. Mirrored stainless steel substrate provides improved signal for Raman spectroscopy of tissue and cells. J Raman Spectrosc. 2017;48:119–25.

44. : ASTM E1840-96 Standard guide for Raman shift standards for spectrometer calibration. ASTM International West Conshohocken, PA.
